# Increased risk of chronic fatigue syndrome in patients with inflammatory bowel disease: a population-based retrospective cohort study

**DOI:** 10.1186/s12967-019-1797-3

**Published:** 2019-02-22

**Authors:** Shin-Yi Tsai, Hsuan-Ju Chen, Chon-Fu Lio, Chien-Feng Kuo, An-Chun Kao, Wei-Shieng Wang, Wei-Cheng Yao, Chi Chen, Tse-Yen Yang

**Affiliations:** 1 Department of Laboratory Medicine, MacKay Memorial Hospital, Taipei, Taiwan; 2 Department of Medicine, Mackay Medical College, New Taipei City, Taiwan; 3 Graduate Institute of Long-Term Care, Mackay Medical College, New Taipei City, Taiwan; 4 Graduate Institute of Biomedical Sciences, Mackay Medical College, New Taipei City, Taiwan; 5 Department of Health Policy and Management, Johns Hopkins University Bloomberg School of Public Health, Baltimore, MD USA; 6 Management Office for Health Data, China Medical University Hospital, Taichung, Taiwan; 7 College of Medicine, China Medical University, Taichung, Taiwan; 8 Institute of Infectious Disease, MacKay Memorial Hospital, Taipei, Taiwan; 9 Department of Anesthesiology and Pain Medicine, Min-Sheng General Hospital, Tao-Yuan, 330 Taiwan; 10 Molecular and Genomic Epidemiology Center, China Medical University Hospital, Taichung, Taiwan; 11 Division of Nephrology, Department of Internal Medicine, Changhua Christian Hospital, Changhua, Taiwan

**Keywords:** Chronic fatigue syndrome (CFS), Myalgic encephalomyelitis (ME), Inflammatory bowel disease, Oxidative and nitrosative stress (O&NS) pathways, Microbiota-gut-brain interactions, Bacterial translocation, Immunoinflammatory pathways

## Abstract

**Background:**

Similarities in the symptoms of chronic fatigue syndrome (CFS) and inflammatory bowel disease (IBD) have been observed as follows: severe disease activity in IBD correlates with severe fatigue, major psychiatric signs, the common use of medication, and bacterial translocation. One of several hypotheses for explaining the mechanisms underlying CFS suggests a similarity to the impaired intestinal mucosa of IBD. “This study investigated the risk of incident CFS among patients with IBD”.

**Methods:**

We conducted a population-based retrospective cohort study by using Taiwan’s National Health Insurance Research Database to evaluate the subsequent risk of CFS in patients with IBD, according to demographic characteristics and comorbidities. The exposure cohort comprised 2163 patients with new diagnoses of IBD. Each patient was randomly selected and frequency matching according to gender and age with four participants from the general population who had no history of CFS at the index date (control cohort). Cox proportional hazards regression analysis was conducted to estimate the relationship between IBD and the subsequent risk of CFS.

**Results:**

The exposure cohort had a significantly higher overall risk of subsequent CFS than that of the control group [adjusted hazard ratio (Christophi in Inflamm Bowel Dis 18(12):2342–2356, 2012) = 2.25, 95%, confidence interval (Aaron and Buchwald in Ann Intern Med 134(9 Pt 2):868–881, 2001; Farraye et al. in Am J Gastroenterol 112:241, 2017) 1.70–2.99]. Further analysis indicated a significantly higher risk of CFS in patients who were male (HR = 3.23, 95% CI 2.12–4.91), were older than 35 years, and had IBD but without comorbidity status, e.g. Cancers, diabetes, obesity, depression, anxiety, sleep disorder, renal disease (HR = 2.50, 95% CI 1.63–3.84) after adjustment.

**Conclusion:**

The findings from this population-based retrospective cohort study suggest that IBD, especially Crohn’s disease, is associated with an increased risk of subsequent CFS.

**Electronic supplementary material:**

The online version of this article (10.1186/s12967-019-1797-3) contains supplementary material, which is available to authorized users.

## Background

Chronic fatigue syndrome (CFS), also called myalgic encephalomyelitis, is not only fatigue. This is a cluster of clinical symptoms which is defined as the presence of unexplainable fatigue lasting more than 6 months and accompanied by four or more of the following symptoms: substantial impairment in short-term memory, tender lymph nodes, sore throat, muscle pain, multiple joint pain without swelling or redness, headache, unrefreshing sleep, and postexertional malaise lasting more than 24 h [1]. A recent study indicated that several infectious agents, such as varicella zoster virus, are linked to CFS [2]. Most importantly, CFS majorly affects productivity. Half of the patients with CFS had discontinued their employment because of fatigue-related symptoms, and the total productivity costs owing to such discontinuation each year represented to the UK economy of approximately £102.2 million [3]. Parents of children with CFS also experienced loss of monthly income (mean = £247) and increased monthly expenditure (mean = £206). Thus, identifying the potential CFS population is crucial for early intervention.

Inflammatory bowel disease (IBD), which includes Crohn’s disease (CD) and ulcerative colitis (UC), is a group of chronic disorders characterized by the chronic inflammation of the gastrointestinal tract. Fatigue can be observed in patients with IBD, and severe disease activity [4] and psychosocial factors [5] have been associated with severe fatigue, even when the disease is in remission [6]. Major psychiatric signs of CFS, such as cognitive impairment [7] and insecure attachment, have been observed in patients with IBD [8]. Interestingly, bacterial translocation is one among the several proposed hypotheses explaining mechanisms underlying CFS [9] and it is also observed in patients with IBD [10]. There has been others working on the correlation between IBD and CFS, and find them coexisting [11–14]. These results raise the speculation of a possible common pathophysiology between IBD and CFS.

Although prolonged fatigue is well known in IBD, there were just some research about CFS comorbid with IBD [15], and no research focusing on the development of myalgic encephalomyelitis or CFS currently. Therefore, we conducted a population-based retrospective cohort study by using the National Health Insurance Research Database (NHIRD) to evaluate the subsequent risk of CFS in patients with IBD.

## Methods

### Data sources

The Taiwan National Health Insurance program provides affordable health care to all residents of Taiwan and covered over 99% of the 23 million Taiwan residents since March 1, 1995 (Database NHIR. Taiwan, http://nhird.nhri.org.tw/en/index.html).

Large computerized databases derived from this system by the National Health Insurance Administration, Taiwan and maintained by the National Health Research Institutes, Taiwan, are provided to scientists in Taiwan for research purposes. The National Health Research Institutes (NHRI) has collected health claims data in a de-identified format and established the NHIRD. We used the Longitudinal Health Insurance Database (LHID), which contained the historical claims data from 1996 to 2011 of one million patients randomly sampled from the entire insured population. Disease history is recorded using International Classification of Diseases, Ninth Revision, Clinical Modification (ICD-9-CM) codes.

### Study population

**Fig. 1:**
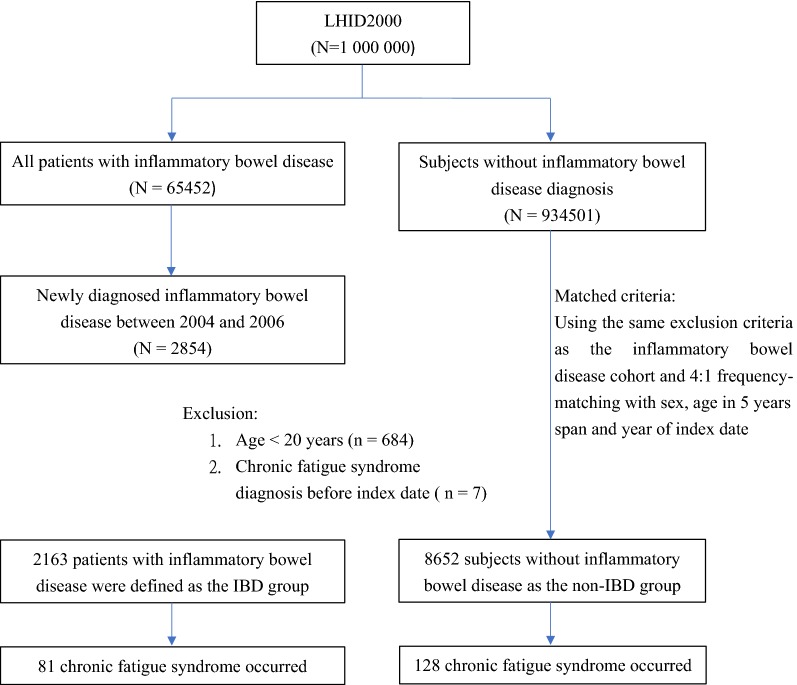
The selection process of the participants in the cohort study

We included patients aged 20 years and older who were newly diagnosed with IBD, including CD (ICD-9-CM 555.0-555.2 and 555.9) and UC (ICD-9-CM 556), between 2004 and 2006 in the IBD group from which we excluded patients with a previous diagnosis of CFS (ICD-9-CM code 780.71) and those with missing age or sex information. The CFS diagnostic criteria has followed that the Fukuda et al. [1] definition of CFS. The date of the first IBD diagnosis was used as the index date. For each IBD patient, four comparisons were randomly selected from the pool of participants without IBD and CFS at the baseline, frequency matched by the year of index date, age (every 5-year span) and sex. In total, 2163 and 8652 patients were included in the IBD and non-IBD groups, respectively. The demographic data included sex and age. We considered cancer (ICD-9-CM: 140-208 from HV), diabetes (ICD-9-CM codes 250), obesity (ICD-9-CM codes 278.0), depression (ICD-9-CM: 296.2, 296.3, 300.4, and 311) [2], anxiety (ICD-9-CM: 300.00), sleep disorder (ICD-9 code 307.4 and 780.5) [16], and renal disease (ICD-9-CM codes 580–589) [17] diagnosed before the index date as preexisting comorbidities (Additional file 2). We used a diagram to illustrate the flow of participants (Fig. 1).

The primary study outcome was the diagnosis of incident CFS. Both groups were followed from the index date until the diagnosis of CFS, withdrawal from the NHI program, or December 31, 2011.

### Statistical analyses

The proportionate distributions of demographic data and comorbidities in the IBD and non-IBD groups were compared and analyzed using the Chi-square test for categorical variables, and the differences were analyzed using the Student t test for continuous variables. The Kaplan–Meier method was used to estimate the cumulative rate of CFS, and the log-rank test was used to examine differences between the survival curves. Cox proportional hazards models were used to assess the independent effects of IBD after adjustment for sex, age, and comorbidities in the model. In addition, we compared the hazard ratio (HR) of CFS between the IBD and non-IBD groups after stratification by sex, age groups, and comorbidity status. We used the SAS software (Version 9.4 for Windows; SAS Institute Inc, Cary, NC) to perform all data analyses, and *P* < 0.05 was considered statistically significant.

### IRB approval

The Ethics Review Board of China Medical University Hospital (CMUH-104-REC2-115) and the Institutional Review Board of MacKay Memories Hospital (16MMHIS074) have approved the study.

## Results

The demographic data and comorbidities of the study population are presented in Table 1. Along with a female predominance (52.07%), the IBD group had a mean age of 47.45 years (standard deviation [SD] [18], 16.52 years). The prevalence of depression, anxiety, sleep disorder, and renal disease was higher in the IBD group than in the non-IBD group.

**Table 1:** Demographic factors and comorbidity of study participants according to inflammatory bowel disease status

Variable	Non-IBD group N = 8652		IBD group N = 2163		*P*-value
	n	%	n	%	
Sex					0.99
Women	4592	53.07	1148	53.07	
Men	4060	46.93	1015	46.93	
Age, years					0.99
20–34	2244	25.94	561	25.94	
35–49	2812	32.50	703	32.50	
50–64	2108	24.36	527	24.36	
≥ 65	1488	17.20	372	17.20	
Means (SD)	47.40	(16.62)	47.45	(16.52)	0.90
Comorbidity					
Cancers	173	2.00	51	2.36	0.34
Diabetes	792	9.15	225	10.40	0.08
Obesity	36	0.42	10	0.46	0.91
Depression	317	3.66	128	5.92	< 0.001
Anxiety	502	5.80	226	10.45	< 0.001
Sleep disorder	1391	16.08	575	26.58	< 0.001
Renal disease	411	4.75	162	7.49	< 0.0001

*IBD* inflammatory bowel disease, *SD* standard deviation

**Fig. 2:**
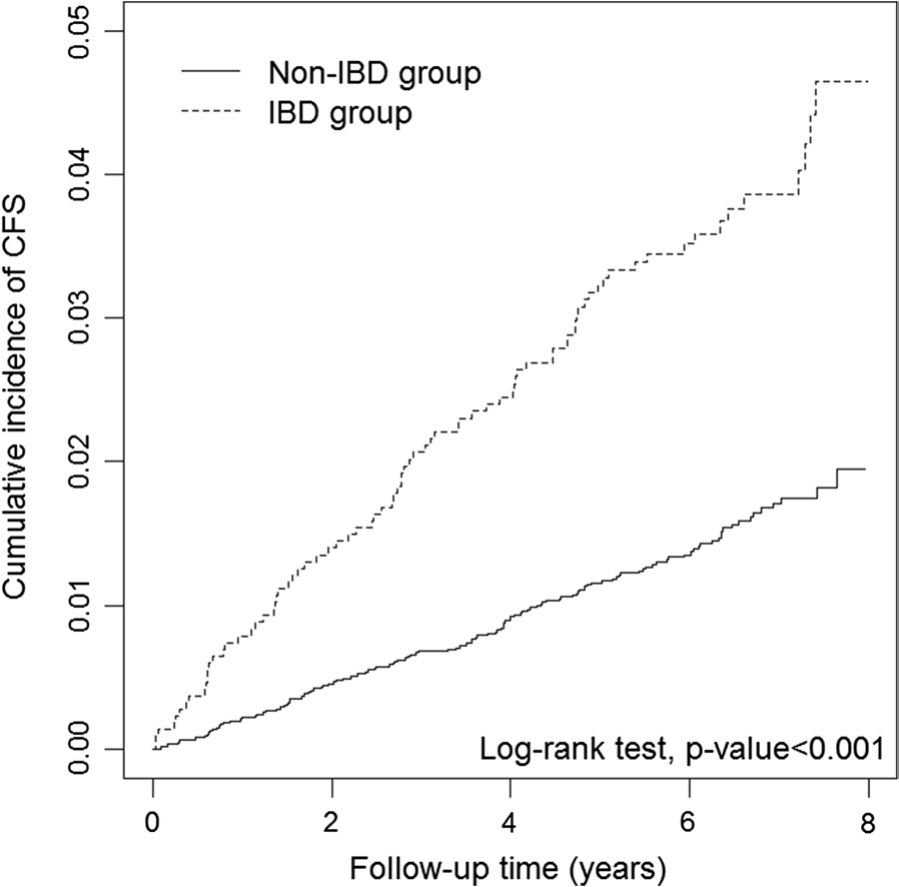
Cumulative incidence curves of chronic fatigue syndrome for groups with and without inflammatory bowel disease

The cumulative incidence curves of CFS according to the IBD status are presented in Fig. 2. The results of the log-rank test revealed that the cumulative incidence of CFS was significantly higher in the IBD group than in the non-IBD group (*P* < 0.001).

The mean (SD) follow-up years of the IBD and non-IBD groups were 6.20 (1.39) and 6.18 (1.37) years, respectively. The overall incidence density rate of CFS in the IBD and non-IBD groups was 6.04 and 2.39 per 1000 person-years, respectively. After adjustment for age, and comorbidities, the risk of CFS was higher in the IBD group than in the non-IBD group (adjusted HR, 2.25; confidence interval [CI], 1.70–2.99; Table 2). The sex-specific analysis revealed that the incidence density rates of CFS in the women and men with IBD were 5.14 and 7.09 per 1000 person-years, respectively, which were higher than those in the women and men without IBD (2.83 and 1.90 per 1000 person-years, respectively). In addition, the risk of CFS was higher in the women and men with IBD than in those without IBD (adjusted HR, 1.67; CI 1.13–2.48; and adjusted HR, 3.23; CI 2.12–4.91, respectively).

**Table 2:** Incidence density rates and hazard ratios of chronic fatigue syndrome according to inflammatory bowel disease status stratified by sex, age, and comorbidity

Variables	IBD						Compared to the non-IBD group	
	No			Yes			HR (95% CI)	
	Event no.	Person-year	IR	Event no.	Person-year	IR	Crude	Adjusted^a^
Overall	128	53,457	2.39	81	13,411	6.04	2.52 (1.91–3.33)***	2.25 (1.70–2.99)***
Sex								
Women	81	28,667	2.83	37	7201	5.14	1.82 (1.23–2.68)**	1.67 (1.13–2.48)*
Men	47	24,790	1.90	44	6210	7.09	3.73 (2.47–5.63)***	3.23 (2.12–4.91)***
Age, years								
20–34	23	14,322	1.61	11	3620	3.04	1.91 (0.93–3.92)	1.83 (0.89–3.78)
35–49	33	17,852	1.85	25	4490	5.57	3.01 (1.79–5.06)***	2.65 (1.56–4.50)***
50–64	41	13,046	3.14	22	3261	6.75	2.15 (1.28–3.60)**	1.81 (1.06–3.07)*
≥ 65	31	8237	3.76	23	2040	11.27	2.97 (1.73–5.09)***	2.79 (1.62–4.82)***
Comorbidity status^b^								
No	62	38,739	1.60	32	8174	3.91	2.43 (1.59–3.73)***	2.50 (1.63–3.84)***
Yes	66	14,718	4.48	49	5237	9.36	2.09 (1.44–3.02)***	2.11 (1.46–3.05)***

*IBD* inflammatory bowel disease, *IR* incidence density rate, per 1000 person-years, *HR* hazard ratio, *CI* confidence interval

* *P *< 0.05, ** *P *< 0.01, *** *P *< 0.001

^a^Model mutually adjusted for age, cancer, diabetes, obesity, depression, anxiety, sleep disorder, and renal disease

^b^Patients with any one of cancers, diabetes, obesity, depression, anxiety, sleep disorder, and renal disease were classified as the comorbidity group

The age-specific analysis revealed that the incidence density rates of CFS increased with age in both groups. In addition, the IBD group had a higher risk of CFS than did the non-IBD group, except for those in the age group of 20 to 34 years. Regardless of the patients’ comorbidity status, the risk of CFS was higher in the IBD group than in the non-IBD group (adjusted HR, 2.50; CI 1.36–3.84 for those without comorbidities; adjusted HR, 2.11; CI 1.46–3.05 for those with comorbidities, respectively).

The further analysis of the two IBD subtypes is presented in Table 3. The patients with CD had a significantly higher risk of CFS than did those without IBD (adjusted HR, 2.27; CI 1.70–3.03) However, compared with the patients without IBD, the patients with UC did not have an increased risk of CFS (adjusted HR, 2.06; CI 0.91–4.69).

**Table 3:** Incidence density rates and hazard ratios of chronic fatigue syndrome in different subgroups

Subgroups	N	Event no.	IR	HR^a^ (95% CI)
Non-IBD group	8652	128	2.39	1.00
IBD group				
Ulcerative colitis	172	6	5.88	2.06 (0.91–4.68)
Crohn’s disease	1991	75	6.05	2.27 (1.70–3.03)***

ICD-9-CM: Ulcerative colitis, 556; Crohn’s disease, 555

*IBD* inflammatory bowel disease, *IR* incidence density rate, per 1000 person-years, *HR* hazard ratio, *CI* confidence interval

*** *P *< 0.001

^a^Model adjusted for age, cancer, diabetes, obesity, depression, anxiety, sleep disorder, and renal disease

## Discussion

A thorough review of relevant research showed that the current study is the first nationwide population-based study to investigate the risk of CFS in patients with IBD. We observed that the risk of CFS was significantly higher in patients with IBD than in the general population. In addition, we identified male sex, advanced age, absence of comorbidities, and CD as the predictors of increased CFS risk. The average age of the sample of newly diagnosed IBD patients was 47.5 years, which is higher than epidemiological studies suggesting peak onset is in the 20s and 30s. This average age may reflect geographic differences [19]. CFS has a multifactorial etiology and several models have been proposed to explain mechanisms underlying CFS, including immunoinflammatory pathways [20], oxidative and nitrosative stress (O&NS) pathways [21], and bacterial translocation [9].

**Fig. 3:**
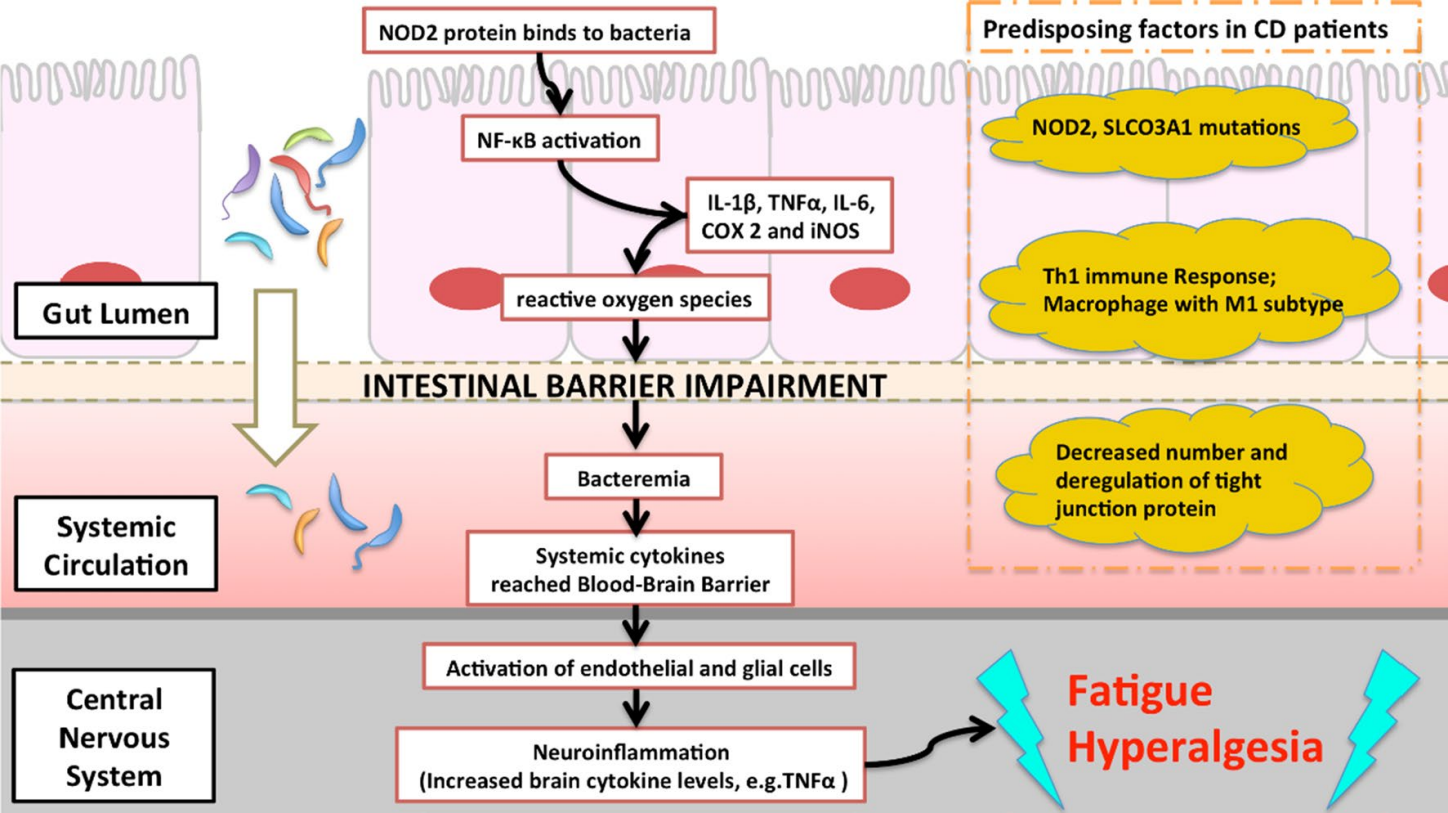
Model of the association between bacterial translocation hypothesis and related traits of Crohn’s disease in the pathophysiology of chronic fatigue syndrome. The extent of the intestinal barrier integrity may have a crucial role in the disease development. Peripheral inflammation can evoke central inflammation through the gut-to-brain pathway, causing major CFS signs

Although the definite pathogenesis of IBD remains unclear, unusual intestinal immune reaction triggered by intestinal flora could lead to inflammation [22] or deficit in the intestinal barrier and bacterial translocation [23]. Noticeably, bacterial translocation has been also proposed as one of the mechanism underlying CFS [9]. This hypothesis could be evidenced by the fact that serum IgA levels against the Lipopolysaccharide (LPS) of enterobacteria were significantly higher in patients with CFS. A study demonstrated the peripheral inflammation is induced by the LPS via binding to the toll-like receptor-4 complex [24]. If there is a mutation of Nucleotide binding oligomerization domain 2 (NOD2), a protein that binds to the peptidoglycan of bacteria resulting in NF-κB activation and inflammatory response, it could lead to CD development [25]. NF-κB is associated with a subjective feeling of fatigue [26] and activation of this pathway is common in both IBD [27] and CFS populations. Moreover, pro-inflammatory cytokines signals could also be relayed to brain by the autonomic nervous system and activated microglia could result in neuroinflammation and increased cytokine levels in brain [28]. There is an association between increased brain Interferon-γ (IFNγ), levels and certain somatic traits such as fatigue and hyperalgesia [29]. The hypothesis of bacterial translocation from the gastrointestinal tract is illustrated as Fig. 3. However, the above hypothesis is one of the possible explanations. Dysbiosis of the gut microbiota and an increased incidence of microbial translocation were suggested to play a principal role in inflammatory symptoms in CFS [30]. Thus, microbiota-gut-brain interactions were indicated essentially in the clinical presentations of a subgroup of patients with CFS [31, 32]. More basic research is warranted before we justify the role of gut-brain inflammation in CFS pathogenesis [18].

We observed that the risk of CFS was higher in patients with CD than in patients without IBD; however, the risk was not higher in patients with UC. Although it may be caused by the underpowered of the data of UC (26.3% power), there are some possible implications from a clinical point of view. The extent of the intestinal barrier integrity may have a crucial role. Firstly, the extent of the inflammation of UC is restricted in colorectal region, and it invades mainly within the mucosa, whereas CD invades different areas of the digestive tract with transmural involvement. On the other hand, a curative operation can be conducted in UC patients, while there is no known cure for Crohn’s disease. To be speculative, the impairment of the intestinal barrier and bacterial translocation may be more severe in CD, and the immune responses exhibited in UC may be less because of the relatively complete integrity of the gut barrier.

It is noteworthy that certain probiotics, such as *Lactobacillus acidophilus*, *Bifidobacterium bifidum* and *Lactobacillus bulgaricus*, and a specific formula diet showed protective effects on the intestinal barrier and decreased rate of bacterial translocation among patients with biliary disease [33]. Future studies can aim to access the response of these therapies in IBD patient with CFS.

The strength of the study obviously is the large number of patients included in both groups (cases and controls). The NHI database of Taiwan provides complete and valid information regarding the demographic characteristics of patients in both the case and control groups. Since we have considered variables, such as sex, age, comorbilities and medical treatment and adjust them individually as well.

Our study has some limitations. First, because of the availability of limited information related to claims data in the NHIRD, we could not further evaluate the effect of biochemical laboratory data and disease severity of patients with IBD. However, this can be investigated by conducting a hospital-based study in which the biochemical laboratory data can be obtained, following which the patients can be stratified into groups according to the severity of their clinical diagnoses. In addition, prescription details are not included in this study. Fatigue has been reported as a side effect to certain medications used in IBD, but we believe that the impact on the incidence rate of CFS is minimal, because the diagnosis of CFS requires not only the long lasting complaints of fatigue but should be “unexplainable” in nature [1]. Theoretically, the diagnoses of IBD and CFS were reliable because this study only included hospitalized patients whose diagnoses were strictly audited for the purpose of reimbursement. Reasonably, specialists should address the issue of adverse events from the use of biologic agents and steroid during the diagnosis. Moreover, the high prevalence of fatigue in IBD is not related to the tapering of steroid [34].

Furthermore, genetic and territorial discrepancies among the different populations should be investigated by conducting additional multinational further studies.

Based on our findings, it’s essential to pay attention not only to the medications applied on the patients with IBD and susceptible CFS, but also to variability in the type and cost of care delivered to patients. Thus, several gastroenterology societies are developing measures to assess quality of care, which should be integrated to quality measures of care in practice [35].

## Conclusion

This study is the first nationwide population-based study to investigate the risk of CFS in patients with IBD. The incidence of CFS, especially CD, was significantly higher in the IBD group than in the non-IBD group. The pilot study finding is essential to provide insights for identifying high-risk patients likely to suffer from CFS and to open a new avenue of research on the intrinsic defects in IBD patients that precipitates CFS. Future studies may aim to access the response of these therapies in IBD patient with CFS. Therefore, immunotherapy to alleviate the state of illness in IBD patients and consequently to improve the patients’ quality of life warrants research (Additional files 1, 2).

## Additional files

**Additional file 1.** The abbreviations and acronyms.

**Additional file 2.** The reported diseases and their ICD-9 codes.
