# Supplementary material for: Increased risk of chronic fatigue syndrome in patients with inflammatory bowel disease: a population-based retrospective cohort study
Source: J Transl Med. 2019 Feb 22;17:55. doi: 10.1186/s12967-019-1797-3 (PMC6387539; doi:10.1186/s12967-019-1797-3)
Supplement: Supplementary file 1 — Additional file 1. The abbreviations and acronyms. [file 12967_2019_1797_MOESM1_ESM.docx]

| CFS | Chronic fatigue syndrome |
| --- | --- |
| IBD | Inflammatory bowel disease |
| HR | Hazard ratio |
| CI | Confidence interval |
| LHID | Longitudinal Health Insurance Database |
| ICD-9-CM | International Classification of Diseases, Ninth Revision, Clinical Modification |
| SD | Standard deviation |
| O&NS | Oxidative and nitrosative stress |
| LPS | Lipopolysaccharide |
| TNF | Tumour necrosis factor |
| NF-κB | Nuclear factor κB |
| NOD2 | Nucleotide binding oligomerization domain 2 |
| PICs | Circulating pro-inflammatory cytokines |
| IFNγ | Interferon-γ |

**Abbreviation and Acronym List**
