# Supplementary material for: Increased risk of chronic fatigue syndrome in patients with inflammatory bowel disease: a population-based retrospective cohort study
Source: J Transl Med. 2019 Feb 22;17:55. doi: 10.1186/s12967-019-1797-3 (PMC6387539; doi:10.1186/s12967-019-1797-3)
Supplement: Supplementary file 2 — Additional file 2. The reported diseases and their ICD-9 codes. [file 12967_2019_1797_MOESM2_ESM.docx]

| Depression | [296.2 - Major depressive disorder single episode](https://icd.codes/icd9cm/2962) |
| --- | --- |
|  | 296.3 - Major depressive disorder recurrent episode |
|  | 300.4 - Dysthymic disorder |
|  | [311 - Depressive disorder, not elsewhere classified](https://icd.codes/icd9cm/311) |
| Anxiety | 300.00 - Anxiety state, unspecified |
| Sleep disorder | 307.4 - Specific disorders of sleep of nonorganic origin |
|  | 780.5 - Sleep disturbances |
| Renal disease | [580 - Acute glomerulonephritis](https://icd.codes/icd9cm/chapter10/580-589) |
|  | [581 - Nephrotic syndrome](https://icd.codes/icd9cm/chapter10/580-589) |
|  | [582 - Chronic glomerulonephritis](https://icd.codes/icd9cm/chapter10/580-589) |
|  | [583 - Nephritis and nephropathy not specified as acute or chronic](https://icd.codes/icd9cm/chapter10/580-589) |
|  | [584 - Acute kidney failure](https://icd.codes/icd9cm/chapter10/580-589) |
|  | [585 - Chronic kidney disease (ckd)](https://icd.codes/icd9cm/chapter10/580-589) |
|  | [586 - Renal failure, unspecified](https://icd.codes/icd9cm/chapter10/580-589) |
|  | [587 - Renal sclerosis, unspecified](https://icd.codes/icd9cm/chapter10/580-589) |
|  | [588 - Disorders resulting from impaired renal function](https://icd.codes/icd9cm/chapter10/580-589) |
|  | [589 - Small kidney of unknown cause](https://icd.codes/icd9cm/chapter10/580-589) |
